# *Epimedium koreanum* Extract and Its Flavonoids Reduced Atherosclerotic Risk via Suppressing Modification of Human HDL

**DOI:** 10.3390/nu11051110

**Published:** 2019-05-18

**Authors:** Jae-Yong Kim, Sang Hee Shim

**Affiliations:** College of Pharmacy, Duksung Women’s University, Seoul 01369, Korea; kjaey0331@naver.com

**Keywords:** *Epimedium koreanum*, des-*O*-methyl-β-anhydroicaritin, high-density lipoprotein, oxidation, glycation, atherosclerosis

## Abstract

Atherosclerosis is the key factor responsible for cardiovascular events, which is a major cause of morbidities and mortalities worldwide. It is well known that high-density lipoprotein (HDL) oxidation and glycation increases the risk for atherosclerosis. *Epimedium koreanum* has been used as a traditional oriental medicine for treating erectile dysfunction, kidney diseases, osteoporosis, and breast cancer. However, no reports on the effects of *E. koreanum* on HDL modification exist. In this study, we investigated the inhibitory effects of *E. koreanum* extract and its eight flavonoids, which are: (1) anhydroicaritin 3-*O*-rhamnoside, (2) β-anhydroicaritin, (3–5) epimedins A-C, (6) epimedoside A, (7) icariin, and (8) des-*O*-methyl-β-anhydroicaritin, against HDL modification. HDLs obtained from pooled human plasma samples were incubated in vitro with *E. koreanum* extract or each compound in the presence of copper sulfate or fructose. The HDL modifications were evaluated by measuring generation of conjugated dienes, production of thiobarbituric acid reactive substances, change in electrophoretic mobility of apoA-I, advanced glycation end products formation, and apoA-I aggregation. Consequently, *E. koreanum* extract and compound **8** suppressed HDL modification through inhibition of lipid peroxidation, apoA-I aggregation, negative charge increase, and AGEs formation. In particular, compound **8** showed more potent inhibitory effect on HDL modification than the extracts, suggesting its protective role against atherosclerosis via inhibition of HDL oxidation and glycation.

## 1. Introduction

Atherosclerosis is a widespread and chronic progressive arterial disease that has been regarded as one of the major causes of morbidities and mortalities worldwide [1]. Numerous studies have demonstrated that high levels of low-density lipoprotein (LDL) and its modification are directly associated with the risk of developing atherosclerosis [2,3]. On the contrary, high level of HDL is well known for its protective role against atherosclerosis. The Framingham heart study, a long-term cardiovascular cohort study, revealed that elevated levels of high-density lipoprotein-cholesterol (HDL-C) were strongly and independently associated with a reduced risk of coronary heart disease (CHD) [4,5], which were also confirmed by further studies [5,6]. In addition, several previous studies have demonstrated that HDL plays a crucial role in the reduction of atherosclerosis risk through reverse cholesterol transport, anti-oxidative, anti-inflammatory, antithrombotic properties, anti-LDL oxidation, and endothelial cell maintenance functions [7,8,9,10]. However, HDL can easily be modified and impaired through a variety of mechanisms including oxidation and glycation and lead to loss of its anti-atherogenic activities [11]. Multiple studies have also reported that oxidized or glycated HDLs induce cytotoxicity and greater risk of cardiovascular and neurological diseases [12,13]. Furthermore, it has also been revealed that modified HDLs are recorded in patients with a variety of diseases, including rheumatoid arthritis, atrial fibrillation, and myocardial infarction [14,15,16]. Therefore, discovery of inhibitors of HDL modification could be a good strategy for the prevention of heart diseases.

*Epimedium koreanum* (*E. koreanum*) Nakai is a perennial and medicinal plant, which is widely distributed in China, Korea, and Japan [17]. In Korea, it has been traditionally used for enhancing erectile function, kidney tonifying, spermatorrhoea, and impotence [18]. Besides, it has also been used widely for the treatment of osteoporosis, immune suppression, cardiovascular diseases, and cancer [18,19,20,21]. *E. koreanum* has been reported to contain diverse constituents including lignans, polysaccharides, and flavonoids [17]. Among them, flavonoids are well-known to exhibit several biological and pharmacological activities including antioxidant, anti-tumor, osteoporosis inhibition, and antiviral effects [19,22,23,24]. In particular, icariin, a major flavonoid from *E. koreanum*, has been reported to have anti-atherogenic effects by modulating some cellular signaling pathways [25,26]. Although *E. koreanum* extract and its flavonoids have various pharmacological roles in the human body, its anti-atherosclerosis function via HDL oxidation and glycation has not been investigated so far. In this study, HDL was separated from plasma of young and healthy male volunteers. Herein, we report the inhibitory effects of ethanol extract of *E. koreanum* and its flavonoids on HDL oxidation and glycation.

## 2. Materials and Methods

### 2.1. Plant Materials and Compounds

The dried aerial parts of *E. koreanum* were purchased from the Herbmana (Gwangju, South Korea) in December 2018. This sample was botanically identified by the corresponding author (Sang Hee Shim). A voucher was deposited at the pharmacognosy laboratory of the College of Pharmacy, Duksung Women’s University (specimen no. NPC 12-6). The dried aerial parts of *E. koreanum* (25 g) were extracted thrice with 1 L of 70% *aq* ethanol (EtOH) for 1 h at 55 °C and the solvents were evaporated in vacuo at 40 °C, yielding the EtOH extract (4 g).

### 2.2. Standards and Reagents 

The standard compounds, anhydroicaritin 3-*O*-rhamnoside (1), β-anhydroicaritin (2), epimedins A-C (3–5), epimedoside A (6), icariin (7), and des-*O*-methyl-β-anhydroicaritin (8), were isolated from the extract of *E. koreanum* by Emeritus professor Sam Sik Kang from Seoul National University. The compounds were identified by nuclear magnetic resonance (NMR) spectroscopy and their purities were assessed by NMR and thin layer chromatography (TLC) analysis before use for this study. Copper sulfate (CuSO_4_), dialysis tubing cellulose membrane, trichloroacetic acid (TCA), 1,1,3,3-Tetramethoxypropane, bovine serum albumin (BSA), and dichlorofluorescein diacetate (DCFH-DA) were purchased from Sigma-Aldrich (St. Louis, MO, USA). Coomassie Brilliant Blue R-250 was purchased from Tokyo Chemical Industry (Tokyo, Japan). 

### 2.3. Isolation of HDL from Human Plasma

Human whole blood samples were obtained from young and healthy male volunteers (IRB no. 2018-007-001). Blood was collected in the Vacutainer (BD sciences, Franklin Lakes, NJ, USA) containing ethylenediaminetetraacetic acid (EDTA, final concentration 1 mM) for each individual. Plasma was separated using high speed centrifugation for 10 min at 4 °C at 3000 rpm (5810R; Eppendorf, Hamburg, Germany). High-density lipoprotein (HDL; *d* = 1.125–1.225 g/mL) was isolated from the plasma via sequential ultracentrifugation in accordance with standard protocols [27]. Plasma was centrifuged for 22 h at 10 °C at 45,000 rpm using a LE-80 (Beckman, CA, USA) at the Instrumental Analysis Center of Duksung Women’s University. Isolated HDL was extensively dialyzed against Tris buffer containing 140 mM NaCl, 10 mM Tris–HCl, and 5 mM EDTA (pH 7.4) for 24 h at 4 °C. Protein quantification of HDL was determined according to the Lowry protein assay with slight modification [28]. 

### 2.4. Measurement of the Formed Conjugated Dienes (CD)

To measure the amounts of dienes formed by lipid peroxidation chain reaction, HDL (200 μg protein/mL) was incubated with CuSO_4_ (final concentration, 10 μM) under the presence of *E. koreanum* extract (final concentrations, 10 and 100 μg/mL, respectively), eight isolated compounds (final concentration, 40 μM), or compound **8** (final concentrations, 10 and 20 μM, respectively) in a medium containing 10 mM phosphate buffer (pH 7.4). During incubation, formation of conjugated dienes was continuously monitored by measuring the absorbance at 234 nm at 37 °C using a Spectra Max 190 spectrophotometer (Molecular Devices, CA, USA) [29].

### 2.5. Measurement of Thiobarbituric Acid Reactive Substances (TBARS)

Native HDL (500 μg protein/mL) with CuSO_4_ (final concentration, 10 μM) in the presence of *E. koreanum* extract (final concentration, 10 and 100 μg/mL, respectively), eight isolated compounds (final concentration, 40 μM), or compound **8** (final concentrations, 10 and 20 μM, respectively) were incubated for 4 h at 37 °C. After oxidation, 0.5 mM EDTA (pH 8.0) was added to terminate the reaction with nitrogen gas. Afterwards, 20% trichloroacetic acid (TCA) was added to HDL samples and reaction mixtures were incubated with thiobarbituric acid (TBA) solution (0.67% TBA in 0.05 N NaOH). The mixture was heated in a water bath at 90 °C for 20 min. The mixed samples were cooled at 4 °C and centrifuged at 3000 rpm for 15 min. After centrifugation, the absorbance of supernatant was measured at 532 nm using a UV–visible spectrophotometer Spectra Max 190 (Molecular Devices, CA, USA) [30]. 

### 2.6. Measurement of Advanced Glycation End Products (AGEs)

To measure the advanced glycation end products (AGEs) formation, HDL (500 μg protein/mL) was incubated with fructose (final concentration, 100 mM) and *E. koreanum* extract (final concentrations, 10 and 100 μg/mL, respectively), eight compounds (final concentration, 40 μM), or compound **8** (final concentrations, 10 and 20 μM, respectively) in 0.2 M sodium phosphate buffer, containing 0.02% sodium azide (pH 7.4) for 72 h at 37 °C. Afterwards, HDL samples were dialyzed for 24 h at 4 °C against 10 mM phosphate buffer (pH 7.4). AGEs of HDL samples were determined by measuring the fluorescence intensity (Ex = 360 nm, Em = 460 nm) using a Synergy 2 multi-mode microplate reader (BioTek, VT, USA) [31].

### 2.7. Measurement of Relative Electrophoretic Mobility (REM)

Electrophoretic mobilities of oxidized HDL by copper was examined using 0.5% agarose gel in TAE buffer (40 mM Tris-Acetate, 1 mM EDTA, pH 8.0) using electrophoretic system (100 V for 40 min). The gels were then fixed using a fixative solution (ethanol: acetic acid: distilled water, 60:10:30, v/v/v) for 30 min to 2 h. After being fixed, the gels were dried in an oven at 80 °C for 1 h, followed by staining with 0.15% Coomassie Brilliant Blue R-250 to visualize the HDL band [32]. 

### 2.8. Measurement of Apolipoprotein A-I (ApoA-I) Aggregation

After oxidation and glycation, each HDL sample was denatured with Laemmli sample buffer and 2-mercaptoethanol (15:1, v/v) at 90 °C for 5 min. 12% and 15% SDS-PAGE was conducted to detect apoA-I aggregation. Then, the gels were stained with 0.15% Coomassie Brilliant Blue R-250 to visualize apoA-I in HDL [33]. 

### 2.9. Measurement of Dichlorofluorescein (DCF) Fluorescence

Dichlorofluorescein diacetate (DCF-DA) dissolved in methanol (final concentration, 2.0 mg/mL) was incubated at room temperature in the dark for 30 min, which released DCFH. Interaction of DCFH with oxidized lipid generated DCF, which produced intense fluorescence. HDLs (500 μg protein/mL) were pre-treated with *E. koreanum* extract (final concentrations, 10 and 100 μg/mL, respectively), eight compounds (final concentration, 40 μM), compound **8** (final concentrations, 10 and 20 μM), or vitamin C (final concentration, 40 μM) for 4 h at 37 °C. Afterwards, HDL samples were dialyzed for 24 h at 4 °C against 10 mM phosphate buffer (pH 7.4). LDL (170 μg protein/mL) was oxidized by CuSO_4_ (final concentration, 0.5 μM) at 37 °C for 4 h in the absence or presence HDL (60 μg protein/mL). Subsequently, copper ions were removed through dialysis. 50 μL of protein mixture was mixed with 10 μL of DCFH and 460 μL of normal saline. Samples were incubated at 37 °C in the dark for 2 h. DCF fluorescence was determined by measuring the fluorescence intensity (Ex = 485 nm, Em = 538 nm) using a Synergy 2 multi-mode microplate reader (BioTek, VT, USA) [34,35].

### 2.10. Statistical Analyses

All data were presented as the mean ± standard deviation (SD), and statistical comparisons between groups were made using paired *t*-tests with the Sigma plot 12.0 statistical software (Systat Software Inc, San Jose, CA, USA). All data were representative of at least 3 independent experiments. *P*-values of less than 0.05 were regarded as significant

## 3. Results

### 3.1. Inhibitory Activities of E. koreanum Extract and Its Flavonoids on HDL Oxidation 

**Figure 1:**
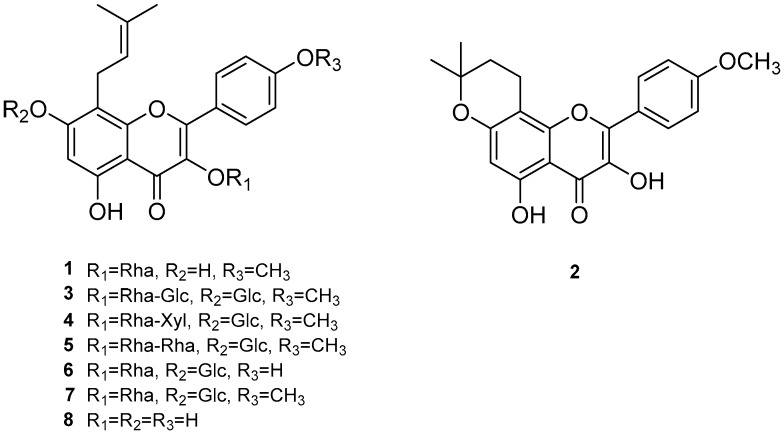
Chemical structures of compounds **1**–**8**; (**1**) anhydroicaritin 3-*O*-rhamnoside, (**2**) β-anhydroicaritin, (**3**) epimedin A, (**4**) epimedin B, (**5**) epimedin C, (**6**) epimedoside A, (**7**) icariin, and (**8**) des-*O*-methyl-*β*-anhydroicaritin.

**Figure 2:**
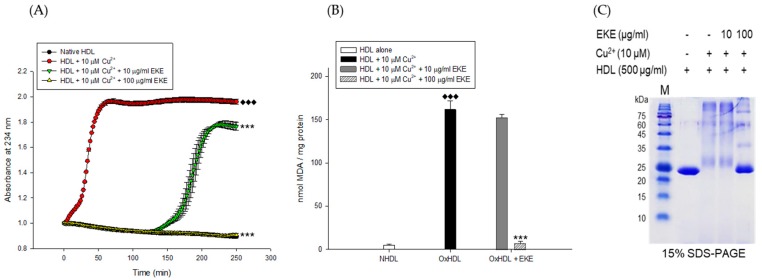
Effects of *E. koreanum* extract (EKE) on Cu^2+^ induced HDL oxidation. (**A**) Continuous monitoring of conjugated diene levels by absorbance at 234 nm (A_234_) during 10 μM Cu^2+^- mediated HDL oxidation in the presence of *E. koreanum* extract (final concentrations, 10 and 100 μg/mL). (**B**) Effects of *E. koreanum* extract (final concentrations, 10 and 100 μg/mL) on TBARS production during HDL oxidation induced by CuSO_4_ for 4 h at 37 °C. (**C**) SDS-PAGE of HDL modified by 10 μM CuSO_4_ for 4 h in the absence or presence of *E. koreanum* extract (final concentration, 10 and 100 μg/mL). These data are expressed as mean ± SD of three independent experiments. ^♦♦♦^, *p* < 0.001 vs. NHDL (native HDL); ***, *p* < 0.001 vs. OxHDL (oxidized HDL).

**Figure 3:**
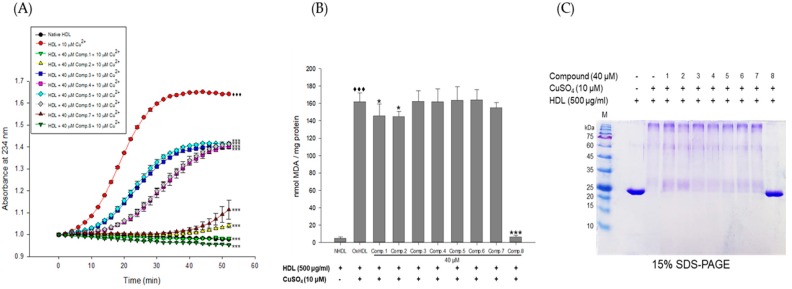
Effects of eight flavonoids from *E. koreanum* on Cu^2+^-induced HDL oxidation. (**A**) Continuous monitoring of conjugated diene levels by absorbance at 234 nm wavelength (A_234_) during 10 μM copper ion mediated HDL oxidation in the presence of the eight compounds (final concentration, 40 μM). (**B**) Effect of the eight flavonoids (final concentration, 40 μM) in TBARS production during HDL oxidation induced by CuSO_4_ for 4 h at 37 °C. (**C**) SDS-PAGE showing HDL modified by 10 μM CuSO_4_ for 4 h in the absence or presence of the eight flavonoids (final concentration, 40 μM). These data are expressed as mean ± SD of three independent experiments. ^♦♦♦^, *p* < 0.001 vs. NHDL (native HDL); *, *p* < 0.01; ***, *p* < 0.001 vs. OxHDL (oxidized HDL).

**Figure 4:**
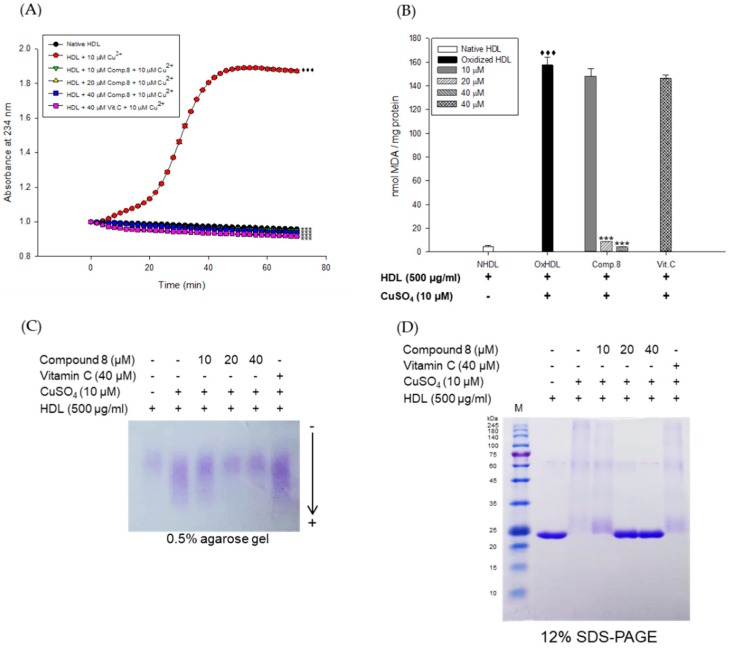
Effect of low concentrations of compound **8** (des-*O*-methyl-*β*-anhydroicaritin) from *E. koreanum* on Cu^2+^-induced HDL oxidation. (**A**) Continuous monitoring of the conjugated diene levels by absorbance at 234 nm (A_234_) during 10 μM copper-ion mediated HDL oxidation in the presence of compound **8** (final concentrations, 10, 20, and 40 μM). (**B**) Effects of compound **8** (final concentrations, 10, 20, and 40 μM) in TBARS production during HDL oxidation induced by CuSO_4_ for 4 h at 37 °C. (**C**) Electrophoretic mobility profile of HDL, which was treated with compound **8** (final concentrations, 10, 20, and 40 μM) in the presence 10 μM copper ion. Oxidized HDL diffused more than native HDL. (**D**) SDS-PAGE of HDL modified by 10 μM CuSO_4_ for 4 h in the absence or presence of compound **8** (final concentrations, 10, 20, and 40 μM). These data are expressed as mean ± SD of three independent experiments. ^♦♦♦^, *p* < 0.001 vs. NHDL (native HDL); ***, *p* < 0.001 vs. OxHDL (oxidized HDL). Vitamin C was used as a positive control.

The inhibitory effects of the *E. koreanum* extract and its eight flavonoids (Figure 1) on HDL oxidation were evaluated by measuring the amounts of CD and TBARS formed, which are the indicators of lipid peroxidation. The extract (at 10 and 100 μg/mL) and its eight flavonoids (at 40 μM) significantly reduced maximum CD formation and further extended the lag time by copper-induced HDL oxidation (Figure 2A and Figure 3A). In addition, 100 μg/mL of *E. koreanum* extract and 40 μM of compounds (**1**–**2**, and **8**) significantly inhibited the formation of TBARS (Figure 2B and Figure 3B). Despite copper-induced oxidation, HDLs treated with 100 μg/mL of *E. koreanum* extract and 40 μM of compound **8** showed similar or even lower levels of CD and TBARS compared with native HDL (Figure 2A,B and Figure 3A,B). In particular, compound **8** showed greater reduction of CD and TBARS formation than other compound (Figure 3A,B). As compound **8** showed strong activities, concentrations lower than 40 μM were used for treatment of the HDLs (at 10 and 20 μM). Low concentrations of the samples also strongly inhibited the formation of CD and TBARS, exhibiting similar levels of CD and TBARS formation to that of native HDL (Figure 4A,B). Vitamin C used as a positive control did not inhibit HDL oxidation at 40 μM and the results were similar to those treated with 10 μM of compound **8**.

The inhibitory effects of *E. koreanum* extract and eight flavonoids on apoA-I aggregation were examined using SDS-PAGE (Sodium dodecyl sulfate-Polyacrylamide gel electrophoresis). The oxidized HDL showed a multimeric pattern of apoA-I, which meant that apoA-I was aggregated by copper ions (Figure 2C). However, HDL treatment with *E. koreanum* extract (at 100 μg/mL) and compound **8** (at 40 μM) inhibited apoA-I aggregation (Figure 2C and Figure 3C). In particular, the apoA-I band of HDL treated with compound **8** (at 40 μM) showed a similar pattern to that of native HDL (Figure 3C). Besides, the low concentration of compound **8** (at 20 μM) also showed similar activity (Figure 4D) to that HDL treated with 40 μM of compound **8**. In an agarose gel electrophoresis, oxidized HDL band was more smeared than that seen in case of native HDL, while the presence of compound **8** (at 20 and 40 μM) recovered copper-mediated changes of positive charge on oxidized HDL (Figure 4C).

### 3.2. Inhibitory Activities of E. koreanum Extract and Its Flavonoids on HDL Glycation

**Figure 5:**
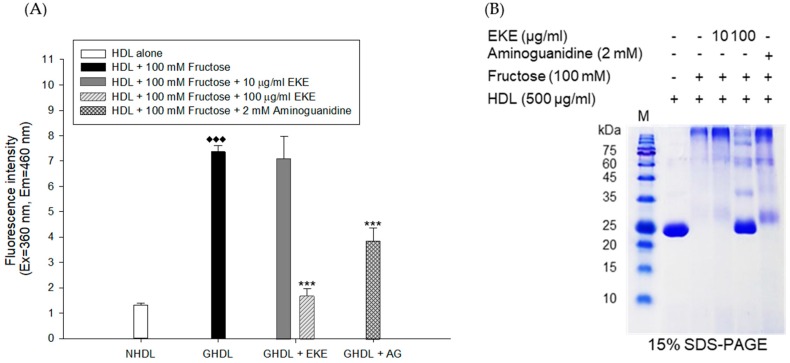
Effect of *E. koreanum* extract (EKE) on fructose induced HDL glycation. (**A**) The formation of AGEs was determined by measuring the fluorescence intensity (Ex = 360 nm, Em = 460 nm). (**B**) SDS-PAGE of HDL modified by 100 mM fructose for 72 h in the absence or presence of *E. koreanum* extract (final concentrations, 10 and 100 μg/mL). These data are expressed as mean ± SD of three independent experiments. ^♦♦♦^, *p* < 0.001 vs. NHDL (native HDL); ***, *p* < 0.001 vs. GHDL (glycated HDL).

**Figure 6:**
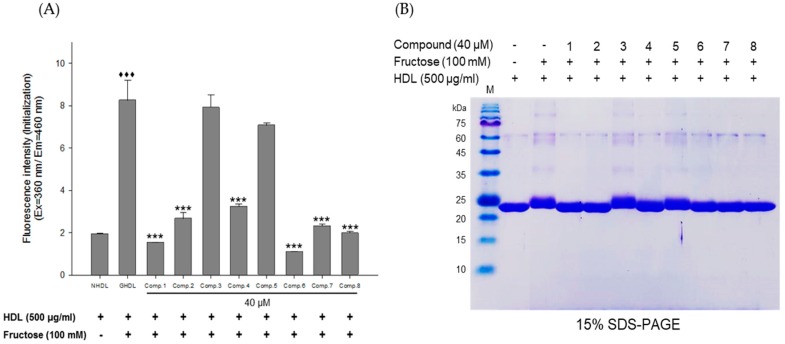
Effects of eight flavonoids from *E. koreanum* on fructose induced HDL glycation. (**A**) The formation of AGEs was determined by measuring the fluorescence intensity (Ex = 360 nm, Em = 460 nm). (**B**) SDS-PAGE of HDL modified by 100 mM fructose for 72 h in the absence or presence of the eight flavonoids (final concentration, 40 μM). These data are expressed as mean ± SD of three independent experiments. ^♦♦♦^, *p* < 0.001 vs. NHDL (native HDL); ***, *p* < 0.001 vs. GHDL (glycated HDL).

**Figure 7:**
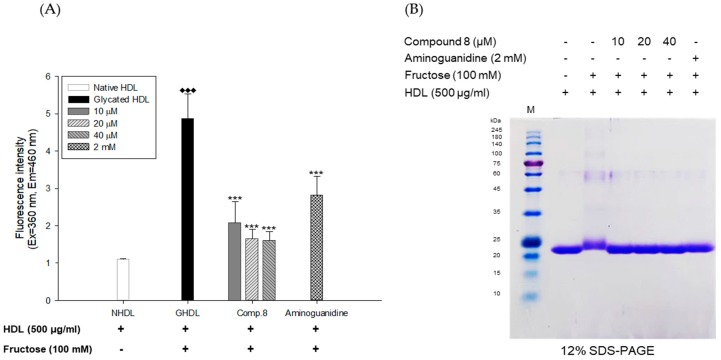
Dose dependent effect of compound **8** (des-*O*-methyl-*β*-anhydroicaritin) on fructose-induced HDL glycation. (**A**) formation of AGEs was determined by measuring the fluorescence intensity (Ex = 360 nm, Em = 460 nm). (**B**) SDS-PAGE of HDL modified by 100 mM fructose for 72 h in the absence or presence of compound **8** (final concentrations, 10, 20, and 40 μM). These data are expressed as mean ± SD of three independent experiments. ^♦♦♦^, *p* < 0.001 vs. NHDL (native HDL); ***, *p* < 0.001 vs. GHDL (glycated HDL).

The inhibitory activities of *E. koreanum* extract and its eight flavonoids on HDL glycation were evaluated by measuring advanced glycation end products (AGEs) and apoA-I aggregation. Glycated HDL induced by fructose showed much higher amount of AGEs than that in native HDL (Figure 5A). However, HDL treated with 100 μg/mL of *E. koreanum* extract and 40 μM of compounds (1, 2, 4, and **6**–**8**) significantly inhibited the formation of AGEs compared to glycated HDL and HDL treated with aminoguanidine (AG) used as a positive control (Figure 5A and Figure 6A). Furthermore, low concentration of compound **8** (10 and 20 μM) also significantly lessened the formation of AGEs (Figure 7A). In the SDS-PAGE analysis, apoA-I in glycated HDL showed multimeric band because of the aggregation caused by fructose, while distinct apoA-I band was observed in case of native HDL (Figure 5B)**.** Despite fructose-induced glycation, HDL treated with 100 μg/mL of *E. koreanum* extract and 40 μM of compounds (**1**, **2**, **4**, and **6**–**8**) reduced apoA-I aggregation (Figure 5B and Figure 6B). Surprisingly, 40 μM of compounds (**1**, **2**, **4**, and **6**–**8**) showed a pattern similar to that of native HDL (Figure 6B). The low concentrations of compound **8** (at 10 μM and 20 μM) also remarkably reduced multimeric apoA-I (Figure 7B). 

### 3.3. Effect of E. koreanum Extract and Its Eight Flavonoids on HDL Function

**Figure 8:**
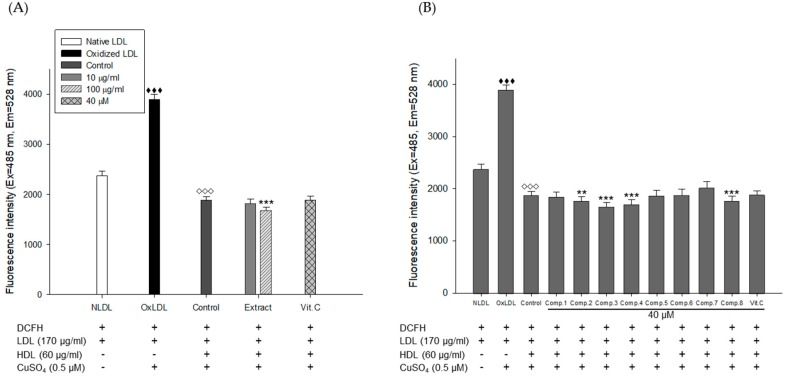
HDL treated with *E. koreanum* extract or its eight flavonoids inhibited ROS production from Cu^2+^-induced LDL oxidation. (**A**) Inhibitory effect of *E. koreanum* extract treated-HDL on LDL oxidation. (**B**) Inhibitory effect of eight flavonoids treated-HDL on LDL oxidation. The formation of DCF was determined by measuring the fluorescence intensity (Ex = 485 nm, Em = 538 nm). These data are expressed as mean ± SD of three independent experiments. ^♦♦♦^, *p* < 0.001 vs. NLDL (native LDL); ^◊◊◊^, *p* < 0.001 vs. OxLDL (oxidized LDL); **, *p* < 0.01, ***, *p* < 0.001 vs. Control.

To evaluate the ability of HDL to inhibit LDL oxidation, a cell-free assay was employed by measuring DCF fluorescence [34]. Oxidized LDL induced by copper-ion significantly increased DCF fluorescence compared to native LDL (Figure 8A). However, it was remarkably reduced by addition of HDL, indicating that HDL inhibited the oxidation of LDL (Figure 8A,B). HDLs treated with *E. koreanum* extract (final concentration, 10 and 100 μg/mL), eight compounds (final concentration, 40 μM), and vit.C (final concentration, 40 μM) also inhibited the LDL oxidation in a similary way to the native HDL (control). In particular, HDLs treated with 100 μg/mL of *E. koreanum* extract and 40 μM of compounds (**1**–**4**, and **8**) showed stronger anti-LDL oxidation activity than the control (Figure 8A,B). 

## 4. Discussion

*E. koreanum* Nakai, mainly comprising flavonoids, has been traditionally used for the treatment of many diseases including cardiovascular diseases [36]. Even though there are some reports that icariin, a major flavonoid from *E. koreanum*, is effective for hyperlipidemia, it remains uncertain whether *E. koreanum* extract and its flavonoids could hinder the oxidation and glycation of HDL or not. In this study, we evaluated the inhibitory effects of *E. koreanum* extract and its flavonoids on the oxidation and glycation of the human HDL. The inhibitory activities against HDL oxidation and glycation were evaluated by measuring the amounts of CDs, MDA, multimeric apoA-I, and AGEs generated from modification of HDL. 

CDs are formed by rearrangement of double bonds in polyunsaturated fatty acids (PUFAs) and primary lipid oxidation products of PUFAs in HDL. The contents of CDs can be quantitatively determined by UV spectroscopy at 234 nm, which have been used to assess the extent of lipid oxidation. Cleavage of these products is known to generate malondialdehyde (MDA). Measuring the formation of CD and MDA is the most commonly employed method to evaluate lipid peroxidation in vitro [31]. CD and MDA have been reportedly generated in substantially large amounts in copper-mediated oxidization of HDL [12,37]. In our results, *E. koreanum* extract and its flavonoids significantly reduced CD formation (Figure 2A and Figure 3A) and inhibited MDA production (Figure 3B). Even low concentrations of compound **8** strongly inhibited the formation of CD and MDA, which were similar to those from HDL treated with the extract and native HDL (Figure 4A,B). These results indicate that *E. koreanum* extract and compound **8** could inhibit lipid peroxidation by reducing CD and MDA formation on copper-mediated oxidized HDL. 

ApoA-I, a major constituent protein of HDLs, is responsible for mediating several beneficial effects in HDL. Modification of apoA-I is directly related to the production of dysfunctional HDL, which has pro-atherosclerotic and pro-inflammatory properties. In a previous study, oxidized HDL induced by CuSO_4_ increased apoA-I denaturation, along with elevating the negative charge on HDL and lipid peroxides in comparison to the native HDL [38]. In the present evaluation, both oxidized HDL and glycated HDL increased formation of multimeric apoA-I (Figure 4D and Figure 5B). In our agarose gel electrophoresis experiment, the band corresponding to the oxidized HDL diffused more than the native HDL, indicating increase of negative charge on the oxidized HDL (Figure 4C). However, HDL treated with *E. koreanum* extract and compound **8** restored the change of charge and inhibited apoA-I aggregation induced by cupric-ion (Figure 2C, Figure 3C, and Figure 4C,D). In glycated HDL, apoA-I aggregation was significantly reduced by treatment of *E. koreanum* extract and compounds (**1**, **2**, **4**, and **6**–**8**) (Figure 5B and Figure 6B). In particular, band for apoA-I of HDL treated with compound **8** showed a pattern similar to that of native HDL, even at low concentrations (Figure 7B). These results suggest that *E. koreanum* extract and compound **8** remarkably inhibit the aggregation of apoA-I and can aid to prevent dysfunctional HDL formation.

AGEs are formed by non-enzymatic reactions between reducing sugars and proteins or lipids. High levels of AGEs have been linked with the development of many diseases such as diabetes, kidney failure, cataracts, Alzheimer’s, and atherosclerosis [38]. Glycated HDL has been reported to significantly increase AGEs formation and reduce activities of paraoxonase [39]. Paraoxonase, an enzyme physically associated with HDL, has an antioxidant activity and prevents oxidation of LDL [40,41]. Recent clinical studies have demonstrated that the glycated HDL-C/HDL-C ratios in patients with metabolic syndrome are higher than that found in controls, suggesting that glycated HDL could be a risk factor to the development of atherosclerosis and diabetes [42]. Our present findings showed that *E. koreanum* extract and its compounds (**1**, **2**, **4**, and **6**–**8**) significantly reduced AGEs formation in fructose-induced glycated HDL (Figure 5A and Figure 6A). In particular, low concentrations of compound **8** (at 10 and 20 μM) reduced AGE formation similar to *E. koreanum* extract (at 100 μg/mL) and compound **8** (at 40 μM). Furthermore, *E. koreanum* extract (at 100 μg/mL) and compound **8** (at 10, 20, and 40 μM) showed a higher potent activity than aminoguanidine (positive control, final concentration, 2 mM) (Figure 7A). These results lead us to believe that *E. koreanum* extract and compound **8** could be considered as an active compound against HDL glycation.

To evaluate the function of HDL to protect LDL against oxidation, a cell-free assay was conducted. The normal HDL significantly decreased the fluorescent signal indicating that the normal HDL strongly inhibited the LDL oxidation. Treatment of *E. koreanum* extract and some flavonoids (**1***–***4**, and **8**) to the HDL also decreased the fluorescent signal even though they did not show dramatic changes compared with the HDL without the extract or compounds. Therefore, this plant and compounds showed additive effects on HDL function against LDL oxidation. 

In this study, *E. koreanum* extract and its flavonoids exhibited potent inhibitory activities against oxidation and glycation of HDL. There are some previous reports that polyphenol-rich dark chocolates and red wine suppressed HDL and LDL modification by lowering oxidative stress levels [43,44]. For a single compound level, genistein, daidzein, luteolin, isohramnetin, quercetin, glabridin, and some anthocyanins increased activities of paraoxonase 1, an enzyme which protects HDL from oxidation, and eventually improved the cholesterol efflux capacity of HDL [45,46,47]. In addition, flavonoid-rich fractions of *Camellia nitidissima* flowers and *Retama sphaerocarpa* fruits showed anti-glycation effects in the glycated-bovine serum albumin (BSA) [48,49]. In our results, the non-glycosylated prenylflavonoid, des-*O*-methyl-β-anhydroicaritin (**8**), was found to show much stronger activities against HDL modifications than the glycosylated prenylflavonoids (**1** and **3**–**7**). In a literature study to compare our results with previously reported ones, limonin, a natural limonoid-type triterpenoid, showed a stronger inhibitory activity on LDL oxidation than its glycoside, limonin 17-β-D-glucopyranoside [50]. Regarding the flavonoid-type compounds, naringenin was also reported to show stronger inhibitory effects on LDL oxidation and glycation than its glycoside, naringin [51], which were quite coherent with our current findings. 

## 5. Conclusions

To the best of our knowledge, this is the first study on inhibitory effects of *E. koreanum* and its flavonoids on HDL oxidation and glycation. Our findings suggest that extract of *E*. *koreanum* and compound **8** (des-*O*-methyl-β-anhydroicaritin) might harbor potential to act as good supplements for the prevention of atherosclerosis via inhibition of HDL oxidation and glycation. Further studies dealing with the mechanistic and in vivo efficacies for compound **8** will be required.
